# Diverse Autonomic Nervous System Stress Response Patterns in Childhood Sensory Modulation

**DOI:** 10.3389/fnint.2020.00006

**Published:** 2020-02-18

**Authors:** Jacquelyn S. Christensen, Heather Wild, Erin S. Kenzie, Wayne Wakeland, Deborah Budding, Connie Lillas

**Affiliations:** ^1^Department of Psychology, Pitzer College, Claremont, CA, United States; ^2^Psychology Program, Portland State University, Portland, OR, United States; ^3^Systems Science Program, Portland State University, Portland, OR, United States; ^4^Harbor-UCLA Medical Center, Los Angeles, CA, United States; ^5^NeuroRelational Framework (NRF) Institute, Pasadena, CA, United States

**Keywords:** autonomic nervous system, sensory modulation, stress response, physiological arousal, automatic processes, complex systems, large-scale network, allostatic load

## Abstract

The specific role of the autonomic nervous system (ANS) in emotional and behavioral regulation—particularly in relation to automatic processes—has gained increased attention in the sensory modulation literature. This mini-review article summarizes current knowledge about the role of the ANS in sensory modulation, with a focus on the integrated functions of the ANS and the hypothalamic-pituitary-adrenal (HPA) axis and their measurement. Research from the past decade illustrates that sympathetic and parasympathetic interactions are more complex than previously assumed. Patterns of ANS activation vary across individuals, with distinct physiological response profiles influencing the reactivity underlying automatic behavioral responses. This review article advances a deeper understanding of stress and the complex stress patterns within the ANS and HPA axis that contribute to allostatic load (AL). We argue that using multiple physiological measurements to capture individual ANS response variation is critical for effectively treating children with sensory modulation disorder (SMD) and sensory differences. We consider the relative contributions of automatic vs. deliberately controlled processes across large-scale neural networks in the development of sensorimotor function and their associated links with arousal patterns and sensory over- and under-responsivity.

## Introduction

Sensory modulation is commonly defined as the ability to regulate and organize reactions to sensations in a graded and adaptive manner (Ayres, 1972; Royeen and Lane, 1991; Parham and Mailloux, 1996; Brown et al., 2019). Yet, the occupational therapy community has grappled with various definitions which bifurcate internal neurophysiological arousal and external behavioral responses to stimuli (Miller et al., 2001, 2007; May-Benson and Schaaf, 2015; Brown et al., 2019). Embedded within the definition of sensory modulation disorder (SMD), a subtype of sensory processing disorder (SPD), is the reference to an individual’s atypical physiological or behavioral responses to everyday stimuli (McIntosh et al., 1999). Physiologically, SMD has historically been considered to reflect disruption in the mechanisms of habituation and sensitization within the central nervous system (CNS; Kandel, 1991). Behaviorally, atypical external responses associated with SMD have been generally categorized as either hyper/over-responsive or hypo/under-responsive as compared to expected response intensity (McIntosh et al., 1999; Miller et al., 2007). However, early observations by Ayres (1963, 2005) posited that children’s disruptions with sensory over-responsivity (SOR) were manifestations of “fight-flight” responses from the autonomic nervous system (ANS) to typical, non-aversive stimulation, suggesting a connection between physiological arousal and behavior. Physiological arousal is simply defined as reflecting a continuum of states of alertness across the sleep-wake cycle (Brazelton, 1973; Barnard, 1999; Oken et al., 2006). It is also more elegantly described as a property distributed across autonomic, sensory, emotional, and motor domains (Pfaff and Banavar, 2007; Mendes, 2016). This latter definition affords a multi-dimensional, non-linear approach to integrating concepts of arousal and sensory responsivity.

The relation between ANS arousal, automatic processes, and sensory responsivity has received increased attention in the sensory modulation literature over the last 25 years (Miller et al., 2009). These research studies attempt to explore connections between external behavioral and internal physiological responses to sensory stimulation, though results are mixed. While children often present clinically with concomitant signs of over-responsivity with heightened arousal and likewise, under-responsivity with lower arousal (Lane, 2002; Schoen et al., 2009), some research finds that physiological arousal and behavioral responsivity are uncoupled (Quas et al., 2000) or mixed (Roubinov et al., 2019).

Multiple contributing factors potentially underlie this inconsistent evidence, including the prevalent use of different, yet singular measures not fully representing the complexity of the stress response system (for full review, see Gomez et al., 2017). Inspired by Gomez et al.’s (2017) larger systematic review, we examine how complex stress and stress recovery models have been researched in isolation, and we review how this fragmentation is paralleled in SMD-focused research. Current neuroscientific approaches featuring large-scale networks, dual-tiered processes and computer modeling offer possibilities to facilitate a more nuanced understanding of physiological variances in arousal and sensory responsivity (Cisek, 2019; Schmahmann et al., 2019). Applying complexity-informed approaches to address the heterogeneity in stress and allostatic load (AL) continuums complement the current shift away from discrete Diagnostic Statistical Manual of Mental Disorders (DSM) diagnostic categories in favor of multidimensional and overlapping processes underlying many disorders. This review article offers recommendations regarding integrated approaches to both SMD research and clinical intervention.

## Stress Models and Arousal in Sensory Modulation Disorder: From Simple to Complex

The following sections describe elements of the ongoing evolution of ANS stress models and their frequent use of limited biomarkers. Many SMD pediatric studies rely solely on parent-completed behavioral checklists to measure sensory responsivity. This review article, however, focuses on SMD studies that also include at least one physiological measure in the context of the Sensory Challenge Protocol (SCP; McIntosh et al., 1999; Miller et al., 1999). This laboratory-based protocol provides a standardized procedure for administering a range of stimuli, which evaluates a child’s physiological arousal reactivity (for reviews of sensory measurements, see Schaaf et al., 2014; Jorquera-Cabrera et al., 2017).

### Sympathetic Nervous System and HPA Axis: Historical Views of Stress and Allostatic Load

Models of stress physiology have historically defined stress response systems as comprising forces of activation and inhibition between two branches of the ANS: the sympathetic nervous system (SNS) and the parasympathetic nervous system (PNS; McEwen, 1998, 2017). The SNS instantiates the fight-or-flight response associated behaviorally with high-intensity motoric mobilization, while the PNS is considered the “rest-and-digest” division of the ANS. Unfamiliar or noxious stimuli can result in simultaneous activation of the SNS and stimulation of the hypothalamic-pituitary-adrenocortical (HPA) axis. Increased amounts of cortisol are subsequently released into the bloodstream, in concert with the restorative response of the PNS, with both facilitating stress recovery (Gunnar and Quevedo, 2007; McEwen, 2007).

Per models grounded in allostatic regulation, when dysregulation prevails within the SNS-HPA axis system, associated neurophysiological responses shift to prolonged activation, inhibition, or both, impacting multiple organ systems (Gunnar and Quevedo, 2007; McEwen, 2007). These subsequent arousal patterns involve temporal dimensions of frequency, duration, and intensity of physiological responses that can go awry, at times accompanied by habituation failures (McEwen, 1998). Resultant wear and tear on the body and brain, impacting both physiological and psychological functioning, is termed AL (see Table 1; e.g., McEwen, 1998, 2017; Goldstein and McEwen, 2002; Berens et al., 2017). These internal arousal patterns often parallel the external behavioral mismatches in grading and regulating the degree and intensity of responses to sensory information that define SMDs (Miller et al., 2007).

**Table 1:** Contributions of key stress models.

ANS-HPA emphasis	Model example	Author/Key citation	Primary measures	Core contributions
SNS HPA	Allostasis/Allostatic Load	McEwen (1998, 2007, 2017)	EDA; PEP (SNS) Cortisol (HPA)	Allostasis—expected stress and stress recovery cycle that allows one to return to baseline Proposed four allostatic load (AL) patterns based upon variation in SNS activation or HPA inhibition. Identified AL patterns underly many disease processes *via* imbalances in the activation (SNS) and inhibition (HPA) wherein the onset of an adult diagnosis is related to long-term immune system wear and tear.
PNS	Polyvagal Theory	Porges (2007); Porges et al. (1996)	RSA or HRV	Identified two PNS vagus nerve branches Offers sequential and hierarchical stages of activation from newer (ventral vagal) to older (dorsal vagal) High PNS activity through HRV measurement associated with better self-regulation and less stress over-reactivity
SNS and PNS	Modes of Autonomic Control	Berntson et al. (1991) Salomon et al. (2000) and Brush et al. (2019)	PEP and HRV PEP and RSA	Proposed nine modes of autonomic control from differing SNS and PNS dynamics—coupled, reciprocal, and uncoupled patterns Found four arousal profile patterns paralleling some patterns within the doctrine of autonomic control—Coactivation and co-inhibition, Reciprocal sympathetic, Reciprocal parasympathetic
SNS and PNS SNS, PNS, and HPA-axis	Adaptive Calibration Model	Del Giudice et al. (2012); Quas et al. (2014)	EDA and RSA PEP, RSA, Cortisol	Originally proposed four patterns of SNS/PNS activity (Sensitive; Buffered; Vigilant; Unemotional) Found six arousal profile patterns in the multi-site study; Moderate reactivity (most common); Parasympathetic-specific reactivity; Anticipatory arousal; Multisystem reactivity; HPA-specific reactivity; Under-aroused

*SNS, Sympathetic nervous system; PNS, Parasympathetic nervous system; HPA-axis, Hypothalamic-pituitary-adrenal axis; EDA, Electrodermal activity; HRV, Heart-rate variability; RSA, Respiratory sinus arrhythmia; PEP, Pre-ejection period*.

Primary biomarkers of SNS activity used in the pediatric stress and SMD literature include a pre-ejection period (PEP) and electrodermal activity (EDA). Derived *via* analysis of electrocardiogram (ECG) data, PEP promotes the use of a singular organ (heart) to examine the synchronicity between the SNS and PNS. Though it is more robust in laboratory settings (Bush et al., 2011, 2016; Schaaf et al., 2015), PEP may be a less sensitive biomarker of SNS compared to other measures in pediatric studies (Roder et al., 2020). Alternatively, EDA measures the conductivity of the skin that results from changes in sweat gland activity (Fowles, 1986) and is well-established as a marker of physiological SNS arousal particularly related to psychological distress (El-Sheikh, 2007; Gatzke-Kopp and Ram, 2018). It is predominantly used to capture variability in physiological sympathetic arousal in the SMD literature (Gomez et al., 2017).

Generally, greater frequency and magnitude of EDA to either all or specific sensory stimulation was observed in the SNS-focused SMD studies reviewed, illustrating that these temporal dimensions were recurrent regardless of diagnosis (see Table 2). While habituation occurred in one study (Schoen et al., 2009), children habituated more slowly in two samples (McIntosh et al., 1999; Su et al., 2010) and fewer children habituated in another (Miller et al., 1999). In addition, a few children with no EDA response to stimulation were reported (McIntosh et al., 1999; Schoen et al., 2009). Most of the reviewed studies found coupling between the reports of external behaviors of SMD and physiological reactivity, and when there was not a match, the higher or lower arousal reactivity remained present. The higher and lower arousal patterns found in SMD implicates sympathetic arousal impairments that may indicate AL conditions, prompting the need for longitudinal naturalistic studies.

**Table 2:** Selected SMD articles by stress response model and physiological patterns.

Study	Sample age	Diagnosis (n)	Physiological measurement	Activation patterns of physiology	Inhibition patterns of physiology
Stress Model: SNS and HPA Axis Focus					
Miller et al. (1999)	4–49	Fragile × Syndrome (15) Fragile × Mutation (25)	EDA (for SNS)	Greater EDA frequency and magnitude; Lower habituation rate	–
McIntosh et al. (1999)	3–9	SMD (19) TYP (19)	EDA (for SNS)	Greater EDA frequency and magnitude; Lower habituation rates	No EDA response to stimulation (*n* = 4)
Mangeot et al. (2001)	5–13	ADHD (26) TYP (30)	EDA (for SNS)	Greater EDA magnitude (early response to sensations)	–
Schoen et al. (2009)	4–15	SMD (31) ASD (38) TYP (33)	EDA (for SNS)	Greater response arousal of EDA (1st trial of sensory stimulation) (SMD); Greater EDA magnitude and amplitude (SMD); Habituation occurred	Lower arousal at baseline (ASD) No EDA response to stimulation found 20–35% of each subgroup
Su et al. (2010)	4–8	SMD (14) TYP (17)	EDA (for SNS)	Greater EDA frequency and magnitude; Slower habituation	–
Miller et al. (2012)	6–12	SMD (37) ADHD (28) SMD and ADHD (12) TYP (30)	EDA (for SNS)	Greater EDA magnitude (SMD vs. ADHD and TYP)	–
Reynolds et al. (2010)	6–12	ADHD w/ SMD (13) ADHD w/o SMD (11) TYP (24)	Salivary Cortisol (for HPA axis)	–	Blunted cortisol response (ADHD w/o SMD)
Lane et al. (2010)	6–12	ADHD (18); TYP (36); ADHD w SOR (21); TYP w SOR (9)	EDA (for SNS) Salivary Cortisol (for HPA axis)	Twice as many non-specific EDA spikes post a challenge, during the recovery phase (ADHD w/ SOR) Elevated cortisol post a challenge (TYP and ADHD with SOR)	–
Stress Models: PNS Focus					
Schaaf et al. (2003)	4–8	SMD (9) TYP (6)	HRV (for PNS)	–	Significantly lower cardiac vagal tone Lower heart period
Schaaf et al. (2010)	5–12	TYP (40); Severe SMD (15); Moderate SMD (13) Borderline SMD (11)	HRV (for PNS)	–	Severe SMD—lower mean vagal tone during baseline, tones, and prolonged auditory stimulation
Stress Models: SNS and PNS Focus					
No studies specific to SMD done at this time with both biomarkers					

*Note: All studies included used Sensory Challenge Protocol (SCP). SMD, Sensory modulation disorder; TYP, Typical; ADHD, Attention-deficit/hyperactivity disorder; EDA, Electrodermal activity; HRV, Heart-rate Variability; SNS, Sympathetic nervous system; PNS, Parasympathetic nervous system; HPA-axis, Hypothalamic-pituitary-adrenal axis*.

Several SMD-focused studies explored the HPA axis, which modulates ANS activity, by including salivary cortisol collection in their protocols. In a small pilot study, SOR was examined as a moderator of HPA activity in children diagnosed with attention-deficit/hyperactivity disorder (ADHD; Reynolds et al., 2010). Children with ADHD and SOR displayed similar cortisol patterns to typically developing children, while children with ADHD without SOR displayed lower, possibly blunted, cortisol responses (Reynolds et al., 2010). While blunted cortisol is frequently observed in children with ADHD (Ma et al., 2011; Pinto et al., 2016), it is also observed in individuals with early adversity (Bunea et al., 2017; Kuras et al., 2017), illustrating the complex relationship between sensory modulation and stress arousal patterns. Emerging models of HPA reactivity also support various trajectories of “typical” daily cortisol patterns (Van Ryzin et al., 2009). In a larger study that did use more than one physiological measure (EDA and cortisol), Lane et al. (2010) found that the combined measures in conjunction with trait anxiety scores were more predictive of children’s SOR scores than any of these indicators alone, supporting the need to use multiple markers to have a more complete picture of arousal and reactivity. Complex variations in cortisol patterns support exploring within-person differences, furthering the investigation of heterogeneity in multifaceted allostatic arousal patterns within SMD (Gatzke-Kopp and Ram, 2018). Stress response models solely considering solely sympathetic and HPA axis activation *via* EDA or cortisol collection are limited in that they fail to capture the complexity of the ANS, including the role of the PNS.

### Parasympathetic Nervous System Focus

The PNS was historically considered to counterbalance SNS activation, conserving energy as the vagus nerve slows heart rate, facilitating digestion by increasing intestinal activity and relaxing sphincter muscles in the gastrointestinal tract (Browning et al., 2017). The Polyvagal Theory describes two branches of the PNS (Porges, 2001, 2007). The first branch of the vagus nerve comprises the myelinated ventral vagal brake, which modulates heart rate to encourage calm engagement with sensory or relational stimulation. The second branch comprises the unmyelinated dorsal vagal brake, which contributes to the freeze stress response and influences under-responsive and less reactive stress patterns. For example, varying degrees of the behavioral shutdown and motoric immobilization are clinically associated with an under-responsive continuum of depression, dissociation, and fainting, including bradycardia (Porges, 2004, 2009).

Measures of PNS activity are typically derived through ECG, and include heart rate variability (HRV) and respiratory sinus arrhythmia (RSA). Controversy exists regarding the interpretation of HRV measurement output given the complexity and nonlinearity of sympathetic and parasympathetic interactions (for full review, see Laborde et al., 2017). Earlier research regarding the implications of poor vagal tone on regulation, including sleep, feeding, self-soothing, and behavioral challenges (Degangi et al., 1991; Porges et al., 1996), supported the shift in SMD research to consider how poor parasympathetic functioning impacts stress vulnerability and SOR, possibly providing better insight to ANS functioning (Schaaf et al., 2003). In a small pilot study aligned with Porges’s research, children with SMD showed significantly lower cardiac vagal tone than typically developing children (Schaaf et al., 2003). In subsequent research, children with severe SMD displayed lower PNS activity than typically developing children during the use of the SCP, including during prolonged auditory stimulation (Schaaf et al., 2010). In children with SMD as compared to typically developing children, parasympathetic reactivity was found to couple with extreme sensory over- and under-responsivity (Schaaf et al., 2003) and poorer adaptive behavior (Schaaf et al., 2010). These results imply that children with SMD are impacted by both a diminished sympathetic system and parasympathetic impairments that contribute to poor arousal and behavioral adaptations to sensations, possibly contributing to AL conditions. Yet, these studies do not include robust integration of the HPA axis, nor direct measurement of the SNS or capture the nonlinearity of the ANS.

### Sympathetic and Parasympathetic Focus

Traditionally, the SNS is thought to cause activation of the physiological structures it innervates, while the PNS inhibits these same structures in a mutually oppositional fashion. The doctrine of autonomic space asserts that the interaction between sympathetic and parasympathetic branches of the nervous system is not solely inhibitory in nature and that autonomic control is dynamic and synchronous (see Table 1; Berntson et al., 1994; Berntson and Cacioppo, 2004). Berntson and Cacioppo (2004) proposed nine possible interactions within patterns of coupled (including coactivation and co-inhibition), reciprocal, and uncoupled activation and inhibition (independent) within SNS and PNS branches (Berntson et al., 1991, 1993; Koizumi and Kollai, 1992). Others exploring patterns within autonomic space using both SNS and PNS biomarkers found combinations of coupled and reciprocal stress response patterns, concluding that standard stress models often fail to capture such variability (Salomon et al., 2000; Rotenberg and McGrath, 2016; Brush et al., 2019).

To date, SMD-focused research has not used multiple measures to track simultaneous SNS-PNS interaction, though related research focused on sensory differences in autism and ADHD populations have used multiple physiological markers with findings that reveal inconsistent stress patterns supporting heterogeneity in ANS-HPA axis functions (Lane et al., 2010; Schaaf et al., 2015).

### Progression Towards Heterogeneity in Stress Response Patterns

Recent stress research examines heterogeneous stress response patterns by including multiple facets of the ANS-HPA axis (Del Giudice et al., 2011; Quas et al., 2014). The adaptive calibration model, based on biological sensitivity to context theory, aimed to capture heterogeneity through four proposed stress response patterns based on measures of SNS, PNS, and HPA reactivity (Del Giudice et al., 2012). Quas et al. (2014) empirically examined this more nuanced picture of stress response patterns *via* secondary data analysis of four independent studies. These data include PEP, HRV, and cortisol collected at baseline and in response to stimulation. This analysis yielded six distinct profiles of stress reactivity, adding complexity to aforementioned coupled, reciprocal, and uncoupled patterns (see Table 1). While some SMD-focused research also attempts to capture categorical differences (e.g., Schaaf et al., 2010), no studies of SMD have yet implemented this latest approach to stress response research by accounting for multiple biomarkers and patterns of stress reactivity in typical and neurodiverse populations. This approach would deepen our understanding of heterogeneity in stress arousal patterns with the potential for recognizing AL conditions existing within SMD.

## Large-Scale Networks and Dual-Tiered Models

While physiologic reactivity does not always correlate directly with the behavioral response, it does provide an indication that internal levels of arousal and stress are connected to emotional, behavioral, social, and health outcomes (LeDoux and Hofmann, 2018). Widely distributed neural networks developed over millions of years across species help manage our continual process of environmental interaction and exposure to sensory information by maximizing automatized processes (Cisek and Kalaska, 2010; Cisek, 2019). Automatic processes and behaviors are those performed implicitly, while deliberate processes and behaviors are those performed explicitly, although these exist on a continuum and are rarely discrete (Boraud et al., 2018; LeDoux and Daw, 2018). Dual-tier models of automatic vs. deliberate processes and behavior in conjunction with large-scale network functions provide further means of conceptualizing the relationship between internal stress physiology, sensory responsivity, and external behavior.

Two large-scale networks have been presented as contributing to the development of automatic or habitual emotional and behavioral responses. Cerebro-cerebellar and Cerebro-striatal-thalamic circuitry are particularly relevant to sensorimotor development, providing essential regulatory functions in information processing across distributed networks, including autonomic, sensorimotor, affective, and cognitive domains (Koziol et al., 2011, 2012; Shine and Shine, 2014; Schmahmann et al., 2019). The cerebellum potentially plays a central role in which processes become automatic and related circuits are thought to contribute to the gradation of rate, rhythm, and force involved in motor or behavioral modulation challenges resulting in “over-shooting” and “under-shooting” target behaviors often seen in occupational and neurological clinical settings (Engel-Yeger, 2019). For example, the slower and lower rates of habituation reported in several SMD-EDA focused studies (see Table 2) can be viewed through this automaticity-relevant large-scale network lens, and it is consistent with the aforementioned definition of SMD as an inability to grade responses to sensation (Ayres, 1972; Royeen and Lane, 1991; Parham and Mailloux, 1996; Brown et al., 2019). Both Cerebro-cerebellar and Cerebro-striatal-thalamic circuitries are active in mobilizing arousal responses to sensations experienced as threatening. Their complex interactions can contribute to sensitization, which is an increase in arousal reactivity with exposure to the same stimuli, as well as the more typically expected habituation, which is a decrease of arousal with repeated exposure. Sensitization can be found underlying multiple diagnostic categories including autism and trauma-related syndromes (De Bellis and Zisk, 2014; Sinclair et al., 2017).

Additionally, theories of generalized arousal of the CNS (Pfaff and Banavar, 2007; Quinkert et al., 2011; Calderon et al., 2016) propose that arousal reactivity, emotional processes (Tops et al., 2017), and sensory responsivity (Deneve and Pouget, 2004; Olcese et al., 2018), in concert with motor activation (Torres and Whyatt, 2018; Wu et al., 2018), can be considered ongoing, parallel, intersecting processes with automaticity. For example, the neurovisceral integration model (NVI; Thayer and Lane, 2000), spans automatic and deliberate processes (Smith et al., 2016), providing emerging neuroanatomical and experimental support (from rodents and primates) for a variety of distributed control networks supporting the integration of autonomic, emotional, attentional, and cognitive information. To best explore the complex, integrated relationships between temporal dynamics across various large-scale networks, nonlinear approaches and computational modeling are used (Wiley et al., 2016; Shine et al., 2019).

## Conclusion

While many stress models call for a more complex view of physiological stress responses, none until recently have described interactions between more than two physiological branches of the ANS-HPA axis (Quas et al., 2014). This fragmentation and associated dominance of singular physiological biomarkers in both stress model-related and SMD-focused research constrain advancement in both fields towards greater complexity and heterogeneity. Large-scale network models offer several possible frameworks capable of managing the highly complex physiological and behavioral aspects of both stresses- and SMD-related research. First, the multiple reactivities and patterns of arousal should be studied in a more complex and coordinated manner. However, in line with earlier reviews (e.g., Rogers and Ozonoff, 2005; Gomez et al., 2017), we highlight the variability in children’s ANS-HPA axis responses to sensory stimuli, regardless of diagnosis. We view this heterogeneity as a natural and expected continuum of arousal occurring across individual nervous systems. Aligning with NIMH Research Domain Criteria (RDoC; Sanislow et al., 2019), SMD can be viewed as an integral aspect of stress response physiology, providing an underlying dimension to join other categorical diagnostic entities formerly considered discrete. This supports work wherein SMD is expanded beyond neurodiverse populations, and considered an essential means of accessing evidence of autonomic dysregulation characteristic of various populations with vulnerable nervous systems, including individuals with prematurity, mental health diagnosis, or early adversity (Shonkoff et al., 2012; Paul-Ward and Lambdin-Pattavina, 2016; Pears et al., 2016; Andersen et al., 2018; Germain, 2018; Machingura et al., 2018; Brown et al., 2019; Mulkey and du Plessis, 2019).

Second, large scale network models emphasize measuring multiple processes and temporal dimensions occurring across physiological biomarkers. Evidence within SMD and stress research suggests that each biomarker, including EDA, PEP, cortisol, and HRV, can display coupled, reciprocal, and uncoupled activation patterns. These patterns occur in varying frequency, intensity, periodicity, rhythm, and duration. These temporal dimensions can match or mismatch associated context resulting in a heightened or dampened stress response. Further study of ANS-HPA axis heterogeneity as potential indicators of AL patterns (McEwen, 1998) requires simultaneous use of three or more physiological markers across multiple time scales in a variety of settings, more closely representing behavior observed outside of laboratory settings. We suggest that future SMD and stress arousal-focused research track both the external behavioral responses and internal physiological reactivity by capturing ANS-HPA axis activation-inhibition in both short-term and longitudinal time scales. As research-quality wearable sensors become more accessible, integrated arousal and SMD studies can move from the laboratory to community settings to further illuminate the variety of internal and external mismatches that can occur in daily occupations. Thus, non-linear dynamical models are most appropriate for managing the varying temporal dynamics related to ANS-HPA axis systems. Complex systems modeling, which strives to portray causal interrelationships within a system, has been used to generate insight into a wide range of biomedical applications (Wittenborn et al., 2016; Kenzie et al., 2018) and could be advantageous. Finally, automatic and deliberate processes from dual-tiered models inform effective treatment planning by supporting the alignment of treatment approaches across distributed systems. Integrating awareness of arousal regulation with sensorimotor-based treatments are necessary, including a promising trend towards decreasing EDA magnitude (e.g., Miller et al., 2007; Bodison and Parham, 2018; Foitzik and Brown, 2018). Sensorimotor-focused treatment strategies can impact a variety of distributed properties and benefit from being coupled with socio-emotional and play-based relational approaches (Greenspan et al., 1998; Bundy et al., 2008; Lillas et al., 2018; Pfeiffer et al., 2018; Roberts et al., 2018; Schaaf et al., 2018; Delahooke, 2019; Porges et al., 2019). This integrated, interdisciplinary lens better addresses sensorimotor over- and under-responsivity in tandem with the arousal and emotional dysregulation related to internal stress responses.

## Author Contributions

JC contributed to the conceptualization, organization, and primary revisions of the manuscript and drafting tables. HW and DB contributed to the manuscript, providing content knowledge. EK and WW contributed to the manuscript. CL took the lead in the theoretical conceptualization, organization, and writing of the manuscript and offering content to tables. JC and CL supervised the collaboration of the project.

## Conflict of Interest

The authors declare that the research was conducted in the absence of any commercial or financial relationships that could be construed as a potential conflict of interest.
